# Identifying the Epileptic Network

**DOI:** 10.3389/fneur.2013.00084

**Published:** 2013-07-01

**Authors:** Mark D. Holmes, Don M. Tucker

**Affiliations:** ^1^Neurology/Regional Epilepsy Center, Harborview Medical Center, University of WashingtonSeattle, WA, USA; ^2^Electrical Geodesics Inc.Eugene, OR, USA

Progress in characterizing the functional networks of the normal human brain is now rapid, with evidence from both regional correlational patterns from functional MRI and fiber tractography from diffusion MRI. Increasingly, the tools of cerebral network analysis are being applied to understand the derangement of specific cortical and subcortical networks in epileptic disorders. In this approach, the clinical manifestations of epilepsy are viewed as the consequence of the pathologies of network dynamics and functional connectivity that may involve abnormal network pathways. Importantly, concepts of epileptic networks are supplanting the older, and more simplistic, notion that epileptic seizures must be either “focal” (or partial) or “generalized” in nature. Rather, seizures can be understood to result from the paroxysmal and pathological activation of specific neuronal connections. The characteristics of these may not fit with conventional assumptions, and could include widespread and bilateral involvement during seizures which classically are considered as focal, or could involve restricted cortical/subcortical regions during some seizures that are typically considered as generalized in nature. We believe that identifying patient-specific epileptic networks will provide critical insights into epilepsy syndromes, and more importantly, these insights will lead the way to novel forms of treatment for affected individuals.

Technological improvements in several fields have contributed to the tools applied to understanding epileptic networks, particularly in neuroimaging (MRI, FDG-PET, fMRI), and in electromagnetic recordings (dense array EEG, MEG). Investigators are also finding that combining these methodologies may have a synergistic effect in regard to enhancing our understanding of the involved cortical networks. In this volume we have assembled contributions from an international group of investigators, each of whom has approached the problem of identifying the epileptic network from somewhat different perspectives. The unifying theme in all cases is the question of how the application of a specific technology, or a simultaneous combination of technologies, may enhance our insight into the recognition of the epileptogenic zone in the resting state.

This book opens with a chapter by Stefan and Lopes da Silva (1), who review the evidence for the concept of epileptic networks. These authors discuss the structure and dynamics of cortical networks, describe how these connections can be analyzed through linear and non-linear methodologies, and outline the dynamics of neuronal networks in the context of combined EEG/MEG and EEG/fMRI signals analysis. They conclude that the resulting network analysis has clear relevance to understanding the nature of seizures occurring with focal cortical dysplasia and with temporal lobe epilepsy. Remarkably, they suggest that an absence seizure, often considered the prototypical generalized seizure, is actually a fast-spreading localized event.

In a similar vein, Leite et al. (2) propose a novel method for linkage of EEG and fMRI signals in network analysis by describing in their report a “transfer function” between these divergent measures. They perform independent component analysis of EEG and extract metrics that express models of EEG-fMRI function from resulting time courses. These metrics are then used to predict fMRI activity and thus the brain regions associated with epileptic activity. The authors illustrate the methodology in a proof of concept report on the application of this function to fMRI-EEG data obtained during both ictal and interictal states in one subject with a hypothalamic hamartoma.

In the next two chapters, by Constable et al. (3) and Weaver et al. (4) the focus is on using resting state fMRI to assess functional connectivity in the human brain, and how this approach can be applied to epilepsy. These two groups describe the functional reorganization that occurs in epilepsy, and the potential that connectivity measures have in identifying a network of seizure-generating tissues. Both groups stress the importance of focal connectivity measures as adjunctive tools in the identification of the epileptogenic zone in patients with refractory epilepsy who are being considered for resective surgery.

On the other hand, Kerr et al. (5) find that the interictal FDG-PET, by visualization of the metabolic changes that take place across the whole brain in epilepsy patients, offer another method to observe abnormal brain networks in the resting state. These authors report that in temporal lobe epilepsy, examination of patterns of metabolic dysfunction may assist in lateralizing the onset of seizures. They report on the development of a computerized assisted diagnostic tool for implementing the metabolic analysis in clinical practice.

Rose et al. (6) studied simultaneous MEG-EEG activity in a series of children with refractory epilepsy. They studied the MEG signals throughout the brain using a beamformer algorithm, and they determined virtual MEG spike locations with a spike detection program. Comparisons of the MEG results with intracranial EEG recordings were conducted both for EEG spikes and for the onset and spread of seizures. By demonstrating similarities with the invasive electrographic findings, the authors conclude that the pattern of interictal MEG findings has the potential to define the distribution of the epileptic network, thereby providing a non-invasive method to analyze abnormal neuronal connections.

Yamazaki et al. (7) have pioneered the ability to simultaneously record 256 channel dense EEG (dEEG) and invasive subdural EEG recordings in temporal lobe epilepsy, thus helping to establish the validity of dEEG recordings. In their chapter in this volume, Yamazaki et al. (7) extend this work to cases of neocortical epilepsy by demonstrating that dEEG, by covering the whole head with sufficient sensor density, can reliably localize epileptiform discharges when compared to invasive studies.

The final two chapters concern the application of analytic techniques to examine abnormal synchronization of the interictal dEEG data to establish the presumptive epileptogenic zone. Song et al. (8) discuss the use of coherence measures in the examination of interictal spikes to determine the extent and distribution of epileptic networks. In their contribution, Ramon and Holmes (9) provide evidence that brief segments of interictal dEEG, free of classical epileptiform patterns, nevertheless may contain stable markers that reveal the likely epileptic network. These markers are identified through analysis of localized patterns of phase synchronization and cross-frequency coupling that appear specific to the epileptogenic region as proven by later intracranial recordings.

The topics covered in this volume present an introduction to the study of identifying epileptic networks. They are only a sample of the many current approaches to cerebral network analysis that could be applied to epilepsy. Nevertheless, we are hopeful that the material presented here will provide encouragement for additional work to clarify – and treat – the pathological dynamics of human cerebral networks in epilepsy.
